# Supplementary material for: Impact and Cost-Effectiveness of Alternative Human Papillomavirus Vaccines for Preadolescent Girls in Mozambique: A Modelling Study
Source: Vaccines (Basel). 2023 Jun 2;11(6):1058. doi: 10.3390/vaccines11061058 (PMC10304296; doi:10.3390/vaccines11061058)
Supplement: Supplementary file 1 [file vaccines-11-01058-s001.zip › vaccines-2218661-supplementary.pdf]

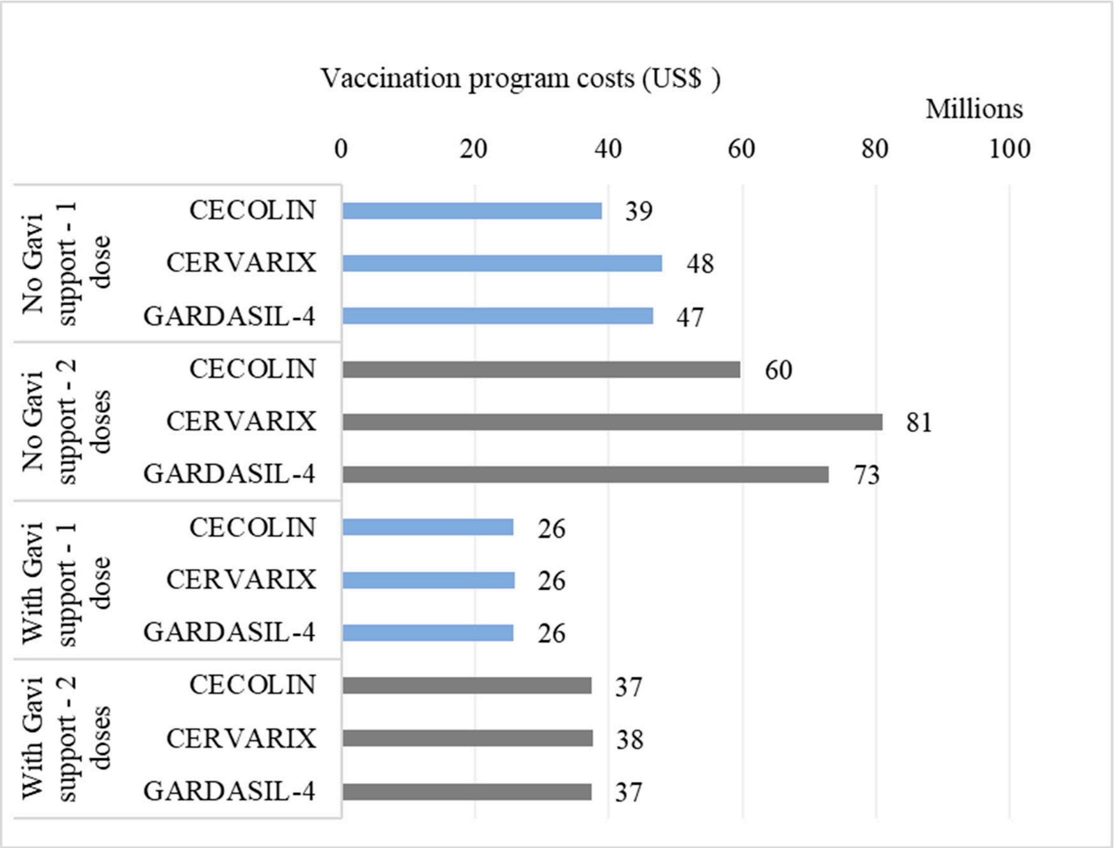


**Figure S1.** HPV vaccination program cost of CECOLIN, CERVARIX and GARDASIL-4, during all the analysis period, 2022 – 2031, including catch-up campaign.

**Table S1.** Deterministic scenarios showing the cost (US$) per DALY averted of vaccination with Gavi support, using CECOLIN, CERVARIX and GARDASIL-4, compared to no vaccine.

| Vaccine | Scenario | Government Perspective |
|---|---|---|
| **CECOLIN** | **Central inputs (US$0.20 per dose)** | **1.9** |
| | Vaccine price = 25% lower (US$ 2.18 per dose) | 20 |
| | Vaccine price = 50% lower (US$ 1.45 per dose) | 13 |
| | Vaccine price = Highest (US$ 2.9 per dose) | 26 |
| | Schedule = 1 dose (efficacy = 2 doses) without cross protection | 0 |
| | Schedule = 1 dose (efficacy = 2 doses) with cross protection | 0 |
| | Lower vaccine coverage | 14 |
| | Higher vaccine coverage | 26 |
| | Discount rate = 10% | 448 |
| | Disease burden rates = Low | 14 |
| | Disease burden rates = High | 0 |
| | Healthcare costs = Low | 10 |
| | Healthcare costs = High | 0 |
| | No Catch-up campaign | 2 |
| **CERVARIX** | **Central inputs (US$0.20 per dose)** | **0** |
| | Vaccine price = 25% lower (US$ 1.32 per dose) | 1 |
| | Vaccine price = 50% lower (US$ 2.64 per dose) | 11 |
| | Vaccine price = Highest (US$ 5.18 per dose) | 27 |
| | Schedule = 1 dose (efficacy = 2 doses) without cross protection | 0 |
| | Schedule = 1 dose (efficacy = 2 doses) with cross protection | 0 |
| | Lower vaccine coverage | 10 |
| | Higher vaccine coverage | 13 |
| | Discount rate = 10% | 544 |

| | | |
|---|---|---|
| | Disease burden rates = Low | 25 |
| | Disease burden rates = High | 0 |
| | Healthcare costs = Low | 1 |
| | Healthcare costs = High | 0 |
| | No Catch-up campaign | 18 |
| **GARDASIL-4** | **Central inputs (US$0.20 per dose)** | **2.8** |
| | Vaccine price = 25% lower (US$ 1.13 per dose) | 11 |
| | Vaccine price = 50% lower (US$ 2.25 per dose) | 21 |
| | Vaccine price = Highest (US$ 4.50 per dose) | 42 |
| | Schedule = 1 dose (efficacy = 2 doses) without cross protection | 0 |
| | Schedule = 1 dose (efficacy = 2 doses) with cross protection | 0 |
| | Lower vaccine coverage | 3 |
| | Higher vaccine coverage | 4 |
| | Discount rate = 10% | 459 |
| | Disease burden rates = Low | 15 |
| | Disease burden rates = High | 0 |
| | Healthcare costs = Low | 11 |
| | Healthcare costs = High | 0 |
| | No Catch-up campaign | 3 |

**Table S2.** Deterministic scenarios showing the cost (US$) per DALY averted of vaccination without Gavi support, using CECOLIN, CERVARIX and GARDASIL-4, compared to no vaccine.

| Vaccine | Scenario | Government Perspective |
|---|---|---|
| **CECOLIN** | **Central inputs (US$2.90 per dose)** | **26** |
| | Vaccine price = 25% lower (US$ 2.18 per dose) | 20 |
| | Vaccine price = 50% lower (US$ 1.45 per dose) | 13 |
| | Schedule = 1 dose (efficacy = 2 doses) Without Cross Protection | 0 |
| | Schedule = 1 dose (efficacy = 2 doses) with Cross Protection | 0 |
| | Lower vaccine coverage | 25 |
| | Higher vaccine coverage | 29 |
| | Discount rate = 10% | 729 |
| | Disease burden rates = Low | 44 |
| | Disease burden rates = High | 14 |
| | Healthcare costs = Low | 34 |
| | Healthcare costs = High | 18 |
| | NO Catch-up campaign | 35 |
| **CERVARIX** | **Central inputs (US$5.18 per dose)** | **32** |
| | Vaccine price = 25% lower (US$ 1.32 per dose) | 1 |
| | Vaccine price = 50% lower (US$ 2.64 per dose) | 11 |
| | Schedule = 1 dose (efficacy = 2 doses) without Cross Protection | 30 |
| | Schedule = 1 dose (efficacy = 2 doses) with Cross Protection | 13 |
| | Lower vaccine coverage | 27 |
| | Higher vaccine coverage | 32 |
| | Discount rate = 10% | 753 |
| | Disease burden rates = Low | 48 |
| | Disease burden rates = High | 17 |
| | Healthcare costs = Low | 51 |
| | Healthcare costs = High | 35 |
| | NO Catch-up campaign | 43 |
| **GARDASIL-4** | **Central inputs (US$4.50 per dose)** | **42.0** |
| | Vaccine price = 25% lower (US$ 1.13 per dose) | 11 |
| | Vaccine price = 50% lower (US$ 2.25 per dose) | 21 |
| | Schedule = 1 dose (efficacy = 2 doses) without Cross Protection | 24 |
| | Schedule = 1 dose (efficacy = 2 doses) with Cross Protection | 23 |
| | Lower vaccine coverage | 40 |
| | Higher vaccine coverage | 45 |
| | Discount rate = 10% | 915 |
| | Disease burden rates = Low | 64 |

| | | |
|---|---|---|
| | Disease burden rates = High | 28 |
| | Healthcare costs = Low | 50 |
| | Healthcare costs = High | 34 |
| | NO Catch-up campaign | 57 |
